# Asthma-chronic obstructive pulmonary disease overlap: Results from a national-multicenter study

**DOI:** 10.5578/tt.202401886

**Published:** 2024-03-26

**Authors:** Gülfem Elif ÇELİK, Ömür AYDIN, Elif ŞEN, Tunçalp DEMİR, Bilun GEMİCİOĞLU, Esen KIYAN, Dilşad MUNGAN, İpek KIVILCIM OĞUZÜLGEN, Mehmet POLATLI, Özlem GÖKSEL, Abdullah SAYINER, Nurhayat YILDIRIM, Füsun YILDIZ, Arzu YORGANCIOĞLU, Atilla Halil ELHAN, Öznur YILDIZ, İlknur BAŞYİĞİT, Şermin BÖREKÇİ, Yavuz HAVLUCU, Gülfer OKUMUŞ, Murat TÜRK, Sevgi SARYAL

**Affiliations:** 1 Division of Immunology and Allergy, Department of Chest Diseases, Ankara University Faculty of Medicine, Ankara, Türkiye; 2 Department of Chest Diseases, Ankara University Faculty of Medicine, Ankara, Türkiye; 3 İstanbul University-Cerrahpaşa, Cerrahpaşa Faculty of Medicine, Department of Chest Diseases, İstanbul , Türkiye; 4 Department of Chest Diseases, İstanbul University, İstanbul Faculty of Medicine, İstanbul, Türkiye; 5 Department of Chest Diseases, Gazi University Faculty of Medicine, Ankara, Türkiye; 6 Aydın Adnan Menderes University Faculty of Medicine, Department of Chest Diseases, Aydın, Türkiye; 7 Department of Chest Diseases, Ege University Faculty of Medicine, İzmir, Türkiye; 8 Department of Chest Diseases, Kyrenia University Faculty of Medicine, Kıbrıs, Turkish Republic of Northern Cyprus; 9 Department of Chest Diseases, Celal Bayar University Faculty of Medicine, Manisa, Türkiye; 10 Department of Biostatistics, Ankara University Faculty of Medicine, Ankara, Türkiye; 11 Department of Chest Diseases, Kocaeli University Faculty of Medicine, Kocaeli, Türkiye; 12 Department of Chest Diseases, Erciyes University Faculty of Medicine, Kayseri, Türkiye

## Abstract

**ABSTRACT**

**
Asthma-chronic obstructive pulmonary disease overlap: Results from a national-multicenter study
**

**Introduction:** *Patients with asthma-chronic obstructive pulmonary disease (COPD) overlap (ACO) have a greater disease burden than those with COPD or asthma alone. In this study, it was aimed to determine the prevalence, risk factors, and clinical features of ACO because there are limited national data in Türkiye.
*

**Materials and Methods:** *The study was conducted in a cross-sectional design in nine tertiary-care hospitals. The patients followed with a diagnosis of asth- ma or COPD for at least one year were enrolled in the study. The frequency of ACO and the characteristics of the patients were evaluated in the asthma and COPD groups.
*

*
This is an open-access article under the terms of the Creative Commons Attribution-NonCommercial License, which permits use, distribution, and reproduction in any medium, provided the original work is properly cited and is not used for commercial purposes (http://creativecommons.org/licenses/by-nc/4.0/).*

©Copyright 2024 by Tuberculosis and Thorax. Available on-line at www.tuberktoraks.org

**Results:** *The study included 408 subjects (F/M= 205/203, mean age= 56.24
*

*
± 11.85 years). The overall prevalence of ACO in both groups was 20.8% (n= 85). The frequency was higher in the COPD group than in the asthma group (n= 55; 33.3% vs. n= 22; 9.8%), respectively (p= 0.001). Patients with ACO had similarities to patients with COPD in terms of advanced age, sex, smoking, exposure to biomass during childhood, being born in rural areas, and radio- logic features. Characteristics such as a history of childhood asthma and allergic rhinitis, presence of chronic sinusitis, NSAID hypersensitivity, atopy, and high eosinophil counts were similar to those of patients with asthma (p< 0.001). The annual decline in FEV1 was more prominent in the ACO group (mean= -250 mL) than in the asthma (mean change= -60 mL) and COPD (mean change= -230 mL) groups (p= 0.003).
*

**Conclusion:** *This study showed that ACO was common among patients with asthma and COPD in tertiary care clinics in our country. ACO should be considered in patients with asthma and COPD who exhibit the abovemen- tioned symptoms.
*

**Key words:** *Asthma; COPD; overlap; ACO
*

**ÖZ**

**
Astım KOAH overlap: Ulusal çok merkezli bir çalışma sonuçları
**

**Giriş:** *Astım-kronik obstrüktif akciğer hastalığı (KOAH) overlap (AKO) olan hastaların hastalık yükü, yalnızca KOAH veya astımı olan hastalara göre daha fazladır. Türkiye’de ulusal veriler sınırlı olduğundan AKO’nun prevalansını, risk faktörlerini ve klinik özelliklerini belirlemeyi amaçladık.
*

**Materyal ve Metod:** *Araştırma, dokuz üçüncü basamak sağlık hastanesinde kesitsel olarak gerçekleştirildi. Çalışmaya astım veya KOAH tanısıyla en az bir yıl takip edilen hastalar alındı. Astım ve KOAH gruplarında AKO sıklığı ve hastaların özellikleri değerlendirildi.
*

**Bulgular:** *Çalışmaya 408 kişi dahil edildi (K/E= 205/203, ortalama yaş= 56,24
*

*
± 11,85 yıl). Her iki grupta da AKO’nun genel prevalansı %20,8 (n= 85) idi. KOAH grubunda sıklık astım grubuna göre daha yüksekti (sırasıyla n= 55;
*

*
%33,3’e karşı n= 22; %9,8) (p= 0,001). AKO’lu hastaların ileri yaş, cinsiyet, sigara kullanımı, çocuklukta biyomas maruziyeti, kırsal bölgede doğmuş olma ve radyolojik özellikler açısından KOAH’lı hastalarla benzerlikleri vardı. Çocukluk çağında astım ve alerjik rinit öyküsü, kronik sinüzit varlığı, NSAID aşırı duyarlılığı, atopi, eozinofil sayısının yüksek olması gibi özellikler astımlı
*

*
hastalarla benzerdi (p< 0,001). FEV1’deki yıllık düşüş AKO grubunda (ortala- ma= -250 mL), astım (ortalama= -60 mL) ve KOAH (ortalama= -230 mL)
*

*gruplarına göre daha belirgindi (p= 0,003).*

**Sonuç:** *Bu çalışma ülkemizde üçüncü basamak kliniklere başvuran astım ve KOAH hastalarında AKO’nun yaygın olduğunu gösterdi. Yukarıda belirtilen semptomları gösteren astım ve KOAH hastalarında AKO düşünülmelidir.
*

**Anahtar kelimeler:** *Astım; KOAH; astım-KOAH overlap; AKO
*

## INTRODUCTION

In recent years, data have accumulated on the coexistence of asthma and chronic obstructive pulmonary disease (COPD) in the same patient. This clinical entity was first noted several years ago and was referred to as the Dutch hypothesis (1). More recently, the Global Initiative for Asthma (GINA) and the Global Initiative for Chronic Obstructive Lung Disease (GOLD) reports labeled it first as asthma COPD overlap syndrome (ACOS) and finally as

asthma COPD overlap (ACO) (2). These patients display features of both asthma and COPD. Currently, a great variety exists in the prevalence rates of ACO (between 0.9 and 20%) in different countries (3-7). This can be attributed to the different diagnostic criteria used and the lack of consensus for ACO diagnosis (8-11).

The importance of ACO is mainly attributed to its poor prognostic features as well as a higher disease burden with lower quality of life (12-14). Therefore,

determining ACO is particularly important to introduce new health policies, which would provide early diagnosis and better disease management. In terms of early diagnosis, ACO is based on the suggestion that a proportion of patients with COPD with asthma-like inflammation and eosinophilia respond to inhaled corticosteroid (ICS) therapy. However, because excessive use of ICS drugs can cause adverse effects in patients with COPD, physicians need to be sure about the diagnosis of ACO to ensure a balance between treatment and adverse effects (15). The determination of airway hyperreactivity, which may be predictive of accelerated forced expiratory volume in one second

(FEV1) decline and increased mortality in patients with COPD, makes the ACO diagnosis an important

issue for preventing future risks (16). Therefore, it is important to determine patients who are at risk for the development of ACO as early as possible to introduce protective measures.

Potential risk factors for ACO have been studied in many studies from many countries. Importantly, these results show that individual risk factors vary among different countries. Considering our country, asthma and COPD are among the most common chronic respiratory airway diseases with high morbidity and burden (17). However, we have insufficient national data on the epidemiology, risk factors, clinical characteristics, and treatment issues in ACO to deal with this disease.

In this study, patients with a diagnosis of asthma or COPD who were followed in specialized centers for diagnosis and management of these diseases were evaluated using a standard approach to determine the prevalence of ACO in asthma and COPD groups, as well as to determine risk factors and clinical features in our country. By doing this, it was aimed to determine predictive factors for the existence of ACO in our patients with asthma and COPD and provide data for policymakers to guide the development of national strategies.

## MATERIALS and METHODS

The study was conducted in a cross-sectional design in nine tertiary care hospitals in our country. The study centers were in Marmara (three centers), Central Anatolia (three centers), and the Egean (three centers) regions. Each center is specialized in asthma and COPD care. The study protocol was approved by

the local ethics committee (Date: July 23th, 2012; Approval number: 12-396-12). Patients who gave informed consent to participate in the study were included.

## The Patient Population

The study included all patients aged over 17 years who had a diagnosis of asthma or COPD and who had been followed up in the study center for at least one year with the relevant diagnosis. Co-existence of other diseases, including bronchiectasis or malignancy, was not considered an exclusion criterion if the patient already had a physician- confirmed diagnosis of asthma or COPD.

Forty patients per study parameter were estimated to show relevant statistical results; and therefore, an equal number of patients from each center with a total of 750 patients was planned to be included in the study. After enrollment, demographics [age, sex, birthplace, educational status, occupation, cigarette smoking, body mass index (BMI)], childhood risk factors (e.g., childhood exposures and childhood diseases), comorbidities, and disease characteristics (e.g., clinical presentations, laboratory findings, pulmonary function tests, chest X-ray, oxygen saturation), primary diagnosis (asthma, COPD, or ACO), and medications were recorded.

Disease activity was determined for asthma and COPD by the number of severe attacks, number of emergency room (ER) visits, hospitalizations, and use of oral corticosteroids in the last year. For patients with yearly records of spirometry, current and past (one year ago) FEV1 levels were also recorded.

The data were mainly gathered from the hospital records. No additional diagnostic or therapeutic interventions were made for the study.

The diagnosis of asthma and COPD was based on current guidelines (18,19). The diagnosis of ACO was made according to the presence of the following (2):

1. Fixed airflow obstruction (post bronchodilatory FEV1 <80% and FEV1/FVC <70%)
2. Variable airway obstruction (positive reversibility testing or positive bronchial provocation tests)
3. Clinical presentations compatible with ACO (presence of atopy, age over 40 years, and smoking history of more than 10 pack-years)

## Statistical Analysis

Statistical analyses were performed using the Statistical Package for the Social Sciences software (SPSS version 16.0; Chicago, Illinois). Numeric values are expressed as mean ± standard error of the mean (SEM), and nominal values are given as numbers (n) and percentages (%). Normality assumption for continuous variables was assessed using the Shapiro- Wilk test. One-way analysis of variance (ANOVA) was used to test differences between three groups for normally distributed continuous variables and Tukey’s honestly significant difference (HSD) test was preferred as the post-hoc test. Differences between three groups for non-normally distributed variables were evaluated using Kruskal-Wallis variance analysis. When the p-value from the Kruskal-Wallis test was statistically significant, multiple comparison tests were used to determine which group differed from the others. Categorical variables were assessed using the Chi-square test. P-values less than 0.05 were considered statistically significant.

## RESULTS

**Study Subjects**

Initially, a total of 610 patients were recorded to have a diagnosis of COPD or asthma. Of these, 408 patients (F/M= 205/203) who had at least one acceptable spirometry performed during the previous year were included in the study and constituted the final study group. There were 225 patients in the asthma group and 165 patients in the COPD group. Eighteen (4.4%) patients were diagnosed as having ACO based on diagnostic criteria. The prevalence of ACO according to guideline criteria was 20.8% (n= 85) in the entire study population. The prevalence was 9.8% (n= 22) and 33.3% (n= 55) in the asthma and COPD groups, respectively.

## Demographics

The ACO group had similar age and sex distributions to the COPD group, but different than those of patients with asthma (p< 0.001, for both) (Table 1).

**Table:** 

**Table 1.** Demographics of the study groups				
	**Asthma**	**COPD**	**Overlap**	**p**
n	205	118	85	
Age (years, mean ± SD)	50.7 ± 11	63.2 ± 8.7	59.7 ± 10.9	<0.001
Sex (F/M)	164/41 (80/20%)	14/104 (11.9/88.1%)	27/58 (31.8/68.2%)	<0.001
Education				0.04
Illiterate	13 (6.4%)	4 (3.4%)	3 (3.5%)	
Primary school	89 (43.4%)	51 (44.1%)	39 (45.9%)	
Secondary school	20 (9.8%)	23 (19.5%)	10 (11.8%)	
High school	42 (20.6%)	27 (22.9%)	17 (20%)	
University	40 (19.6%)	12 (10.2%)	16 (18.8%)	
BMI				<0.001
Lean-Normal	41 (20%)	50 (42.4%)	26 (30.6%)	
Overweight	71 (34.6%)	46 (39%)	32 (37.6%)	
Obese	93 (45.4%)	22 (18.6%)	27 (31.8%)	
Occupation				<0.001
Housewife	104 (50.7%)	6 (5.1%)	18 (21.2%)	
Working at any job	44 (21.5%)	5 (4.2%)	7 (8.2%)	
Retired	32 (15.6%)	84 (71.2%)	45 (52.9%)	
Other	25 (12.2%)	23 (19.5%)	15 (17.7%)	
Smoking status				<0.001
Non-smokers	145 (70.7%)	12 (10.1%)	23 (27.1%)	
Ex-smoker	49 (23.9%)	89 (75.4%)	51 (60%)	
Current smoker	11 (5.4%)	17 (14.4%)	11 (12.9%)

The patients with COPD or ACO were older and mostly male. Patients with ACO and COPD had a higher pack-year smoking history than patients with asthma. Obesity rate was higher among patients with asthma (p< 0.001) (Table 1).

## Childhood exposures/conditions and diseases

The rate of the patients with ACO who were born in rural areas was similar to those with COPD but higher than that of asthmatics (p= 0.04) (Table 2). Patients with COPD and ACO were also more likely to have lived with farm animals and/or pets during childhood than those with asthma (p= 0.001]). Exposure to tobacco smoke during childhood was high, around 65% for each group, with no differences between the groups. The patients with COPD and ACO had more exposure to biomass during childhood than those with asthma (p= 0.019). Regarding childhood diseases, a history of childhood asthma was more frequent in patients with asthma and ACO than in patients with COPD (p= 0.01). Measles was more common among patients with asthma than in those with COPD or ACO (p= 0.021). Patients with ACO tended to have similar histories of allergic rhinitis to patients with asthma, and a history of childhood pneumonia similar to that of patients with COPD (Table 2).

## Comorbidities

Patients with ACO had similar rates of chronic sinusitis, nasal polyps, and non-steroidal anti- inflammatory drug (NSAID) hypersensitivity to patients with asthma, and the rates of ischemic heart diseases were comparable to those of patients with COPD. Hypertension, bronchiectasis, anxiety, and depression were similar across all groups (Table 3).

## Clinical presentations

The frequency of symptoms of phlegm (COPD: n= 58, 49.2% vs. ACO n= 38; 44.7%), dyspnea on

exertion (COPD: n= 76, 64.4% vs. ACO n= 50, 58.8%), and progressive dyspnea (COPD: n= 73, 61.9% vs. ACO n= 51, 60%) were similar in patients with COPD and ACO and higher than those with asthma (phlegm: n= 39, 19%; dyspnea on exertion: n= 99, 48.3%; progressive dyspnea: n= 49, 23.9%)

(p< 0.001, p= 0.015, and p< 0.001, respectively).

## Laboratory findings

Atopy rates as well as eosinophil levels of the patients with ACO were similar to those with asthma, whereas hemoglobin and hematocrit levels were similar in those with COPD (Table 4). In pulmonary function tests, the degree of airway obstruction and airway trapping were similar in patients with COPD and

**Table:** 

**Table 2.** Childhood risk factors and diseases of the study groups					
	**Asthma**	**COPD**		**ACO**	**p**
**Childhood Conditions/Exposures**					
Birthplace					
Urban	86 (42.4%)	38 (32.2%)	26	(30.6%)	0.04
Rural	117 (57.6%)	80 (67.8%)	59	(69.4%)	
Breastfeeding	195 (95.6%)	109 (92.4%)	81	(95.3%)	>0.05
Attendance to preschool	10 (4.9%)	3 (2.6%)	2	(2.4%)	>0.05
Pet at home	69 (34%)	58 (49.2%)	27	(31.8%)	0.011
Childhood exposure to ETS	135 (66.5%)	79 (66.9%)	59	(67.2%)	>0.05
Exposure to biomass	47 (23.2%)	38 (32.2%)	33	(38.8%)	0.019
**Childhood Diseases**					
Childhood asthma	13 (6.4%)	0 (0)	7	(8.2%)	0.011
Childhood eczema	6 (2.9%)	1 (0.8%)		0 (0)	>0.05
Childhood food allergy	4 (2%)	0 (0)		0 (0)	>0.05
Childhood allergic rhinitis	27 (13.2%)	1 (0.8%)	3	(3.5%)	<0.001
Measles	57 (27.9%)	18 (15.3%)	16	(18.8%)	0.021
Tuberculosis	2 (1%)	6 (5.1%)	4	(4.7%)	>0.05
Pneumonia	15 (7.4%)	26 (22.2%)	11	(12.9%)	0.001

**Table:** 

**Table 3.** Comorbid diseases and disorders of the study groups						
	**Asthma**		**COPD**		**ACO**	**p**
**ACO: Resembling asthma**						
Chronic sinusitis	43 (21%)	10	(8.5%)	11	(42.9%)	0.009
Nasal polyps	10 (4.9%)	1	(0.8%)	10	(11.8%)	0.002
NSAID hypersensitivity	18 (13.7%)	2	(1.7%)	7	(8.3%)	0.002
**ACO: Resembling COPD**						
Ischemic heart disease	11 (5.4%)	22	(18.6%)	15	(17.6%)	<0.001
**Higher in COPD**						
Heart failure	5 (2.5%)	11	(9.3%)	3	(3.5%)	0.016
Lung cancer	0 (0)	3	(2.5%)		0 (0)	0.024
**Higher in Asthma**						
Diabetes mellitus	39 (19%)	14	(11.9%)	5	(6.9%)	0.01
Thyroid disorders	42 (20.5%)	2	(1.7%)	2	(2.4%)	<0.001
Allergic rhinitis	92 (44.9%)	5	(4.2%)	7	(8.2%)	<0.001
Gastroesaphagial reflux	66 (32.2%)	19	(16.1%)	5	(5.9%)	<0.001
Food allergy	14 (6.8%)	1	(0.8%)	1	(1.2%)	0.01
Urticaria	17 (8.3%)	2	(1.7%)		0 (0)	0.002
Higher in ACO						
Tuberculosis	2 (1%)	2	(1.7%)	6	(7.1%)	0.008
**Similar in All Groups**						
Hypertension	65 (31.7%)	41	(34.7%)	33	(38.8%)	>0.05
Bronchiectasis	4 (2%)	3	(2.5%)	4	(4.7%)	>0.05
Anxiety	7 (3.4%)	1	(0.8%)	3	(3.55)	>0.05
Depression	10 (4.9%)	5	(4.2%)	5	(5.9%)	>0.05

ACO and were more severe than in those with asthma (Table 4). In general, the radiologic findings in patients with ACO were concordant with COPD. The most common findings shared by patients with COPD and ACO were hilar enlargement, bronchiectasis, emphysema, and bronchial wall thickening (Table 5).

## Disease activity

The number of exacerbations, admission to the ER due to asthma, and use of systemic corticosteroids due to asthma in the last year were similar in all groups. However, patients with ACO were hospitalized more frequently than those with asthma and COPD (p= 0.029) (Figure 1). The annual decline

in FEV1 was more prominent in the ACO group (mean= -250 mL) than in the asthma (mean change=

-60 mL) and COPD (mean change= -230 mL) groups (p= 0.003) (Figure 2).

## Medications

Long-acting beta-agonists (LABA), short-acting anticholinergics, long-acting anticholinergics, and oral theophyllines were used at similar rates in ACO and COPD and at lower rates in asthma, whereas the use of leukotriene receptor antagonists and nasal corticosteroids were at similar rates in ACO and asthma, but higher than in COPD. Fixed combination inhaled corticosteroid (ICS) and LABA use was higher in patients with asthma (Table 6).

## DISCUSSION

The results of this study highlighted that ACO existed in a significant number of patients with obstructive airway disease, particularly those with COPD in tertiary care clinics in our country. The patients with ACO had childhood exposures similar to both asthma and COPD. In this sense, childhood risk factors of ACO were being born in rural origins and exposure

**Table:** 

**Table 4.** Laboratory evaluation of the study groups				
	**Asthma**	**COPD**	**ACO**	**p**
Positive prick test (n, %)	97 (58.1%)	6 (23.1%)	16 (61.5%)	0.003
Total IgE level (kU/L)	221.2 ± 367	185.5 ± 301	268.4 ± 304	>0.05
Eosinophil count (count per mm3)	302.7 ± 350	190.7 ± 133	322.3 ± 315	>0.05
Eosinophil (%)	3.9 ± 5.1	2.1 ± 1.5	3.7 ± 4.4	0.027
FEV1 (L)	2.19 ± 0.69	1.53 ± 0.66	1.36 ± 0.63	<0.001
FEV1 (%)	86.4 ± 21.4	54.5 ± 20.6	49.5 ± 17.39	<0.001
FEV1/FVC	74.6 ± 8.9	54 ± 11.5	52.8 ± 10.8	<0.001
Inspiratory capacity	2.5 ± 0.85	2.31 ± 0.93	2.36 ± 0.81	>0.05
FRC (L)	2.47 ± 0.92	4.2 ± 1.7	4.46 ± 1.4	<0.001
FRC (%)	86.32 ± 22.4	141.4 ± 42	138 ± 36	<0.001
TLC (L)	5.4 ± 1.12	7 ± 1.3	6.9 ± 1.3	<0.001
TLC (%)	107.6 ± 18.5	112.8 ± 21.34	113.9 ± 16.5	>0.05
Post bronchodilator FEV1 (L)	2.3 ± 0.7	1.6 ± 0.7	1.6 ± 0.6	<0.001
Post bronchodilator FEV1 (%)	92.6 ± 20.22	57.07 ± 22	59.2 ± 18.2	<0.001
Post bronchodilator FEV1/FVC	77.5 ± 8.7	54.7 ± 12.9	54.9 ± 10.5	<0.001
FEV1 reversibility (L)	159.5 ± 143	86.2 ± 77.9	293.2 ± 168	<0.001
FEV1 reversibility (%)	7.6 ± 6.9	5.04 ± 3.76	22.18 ± 11.2	<0.001
PaO2 (mm Hg)	95.9 ± 3.7	92.08 ± 4.6	93.1 ± 5.3	<0.001
PaCO2 (mm Hg)	36.3 ± 3.4	40.4 ± 6.6	40.5 ± 10.1	<0.001
6-minute walk distance (m)	439 ± 122.6	401 ± 151	439 ± 121	>0.05

**Table:** 

**Table 5.** Radiographic features of the study groups				
	**Asthma**	**COPD**	**Overlap**	**p**
**Chest X-ray**				
Hyperinflation	18 (9.2%)	72 (61%)	33 (38.8%)	<0.001
Bronchiectasis	9 (4.6%)	13 (11%)	10 (11.8%)	0.046
Hilar enlargement	11 (5.6%)	31 (26.3%)	16 (18.8%)	<0.001
**Computed Tomography of the Thorax**				
Emphysema	4 (9.5%)	37 (75.5%)	26 (70.3%)	<0.001
Bullae	7 (16.7%)	12 (24.5%)	9 (24.3%)	>0.05
Pulmonary artery enlargement	0 (0%)	12 (24.5%)	6 (16.2%)	0.003
Bronchial wall thickening	4 (9.55)	26 (53.1%)	13 (35.1%)	<0.001
Mosaic perfusion	7 (16.7%)	3 (6.1%)	3 (8.1%)	>0.05
Bronchiectasis	9 (29%)	19 (44.2%)	13 (38.2%)	>0.05

to biomass during childhood; having pets at home and childhood pneumonia were risk factors of COPD, and childhood asthma and allergic rhinitis were risk factors of asthma. Regarding clinical features of ACO, there were again similarities to both asthma and COPD. Patients with ACO had advanced age and male sex like those with COPD, and atopy

and comorbidities such as chronic sinusitis, nasal polyps, and NSAID hypersensitivity similar to those with asthma. The pattern of progressive airway obstruction in ACO like COPD was obvious in clinical presentations and pulmonary functions. In this sense, the degree of airway obstruction was particularly similar to COPD but higher than in

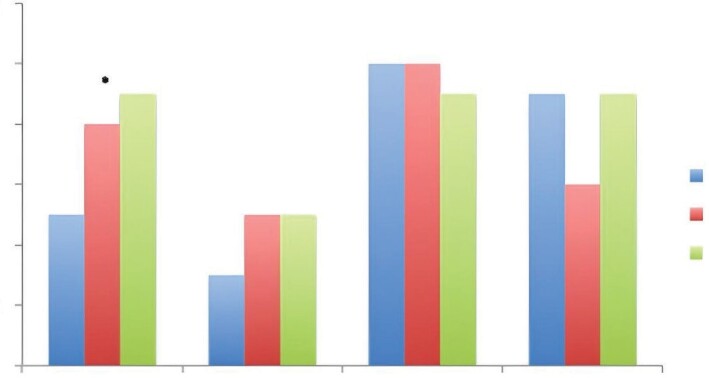

**Figure 1.** Disease activity in the last 12 months in the study groups. Values are given mean ± SD.

*p: 0003 (higher in ACO than in asthma).

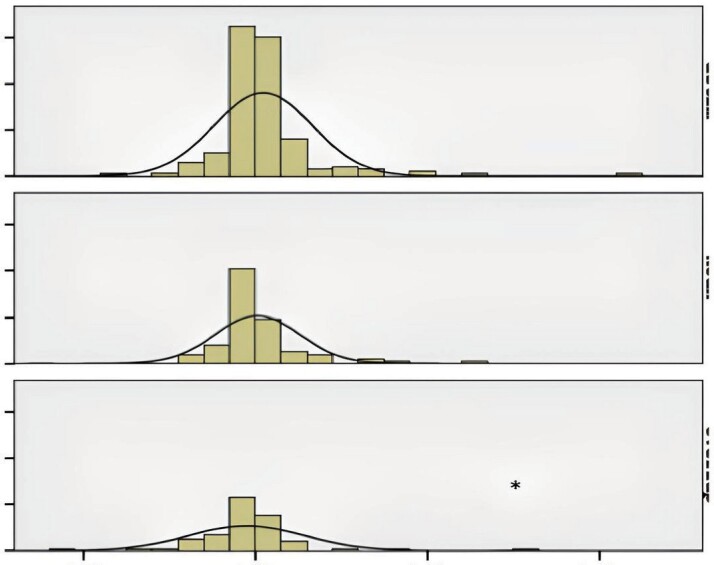
** Figure 2.** Annual FEV1 change pattern in the study groups.

asthma. Annual FEV1 decline was also the highest in ACO among the three groups. Radiologic features also suggested airway obstruction.

Previous studies have reported population prevalence rates for ACO between 0.9% and 20% (3-7). This variability can be explained by differences in the patient populations studied, diagnostic criteria used, and methodologies of the studies. A study that was based on Gene-Environment Interactions in Respiratory Diseases study data showed an ACO prevalence of 1.6-4.5% in the adult general population aged 20-84 years (20). In some previous studies, it has been found that women were more likely to

report ACO when compared with men (21,22). On the other hand, studies performed in certain risk groups have shown higher prevalence rates. As such, the COPD Gene study has shown that 13% of patients with COPD had a diagnosis of ACO (23). Similarly, ACO prevalence has been reported as 24.3% in severe asthmatics (24). The prevalence of ACO has not previously been studied systematically in our country. In a single study performed on 338 patients with asthma, 11% of patients have been shown to have ACO (25). Our results represent the first multicentered data in our country. In general, ACO prevalence was found as 20.8% across the entire population, particularly higher in the COPD

**Table:** 

**Table 6.** Medication use for upper and lower airway diseases of the study groups					
**Medications**	**Asthma**	**COPD**		**ACO**	**p**
**For Lower Airways**					
Inhaled corticosteroid (ICS)	39 (19.3%)	31 (26.3%)	23	(27.7%)	>0.05
Long-acting inhaled beta 2 agonists (LABA)	3 (1.5%)	29 (24.6%)	20	(23.8%)	<0.001
Short-acting inhaled beta 2 agonists	113 (55.4%)	66 (55.9%)	38	(45.2%)	>0.05
Combination of ICS-LABA	165 (80.9%)	78 (66.1%)	60	(70.6%)	0.009
Short-acting anticholinergics	6 (3%)	39 (33.1%)	13	(15.5%)	<0.001
Long-acting anticholinergics	4 (2%)	94 (79.7%)	53	(63.1%)	<0.001
Oral theophylline	6 (3%)	16 (13.6%)	18	(21.7%)	<0.001
Anti-Leukotrienes	101 (49.8%)	5 (4.2%)	17	(20.2%)	<0.001
**For Upper Airways**					
Intranasal corticosteroids	52 (25.6%)	4 (3.4%)	9	(10.7%)	<0.001
Oral antihistamines	46 (22.7%)	0 (0%)	7	(8.3%)	<0.001

group (33.3%). However, the prevalence of ACO among asthmatics was lower (9.8%) and comparable with previous data from our country (5,25). Similar to previous reports, the prevalence was found to be related to advanced age in our group (6,26). In contrast to many trials, the prevalence was higher in men in our study, which may be explained by the higher frequency of ACO among patients with COPD in our study group (10). Considering the high frequency of ACO in the COPD group (approximately one in three patients), physicians who follow up with patients with COPD should be aware of the possibility of the presence of ACO.

We believe that one of the most important findings of this study is that childhood risk factors appear to be relevant for predicting ACO development in at-risk groups. Briefly, if a patient with asthma or COPD has particular risk factors during childhood for each disease, the development of ACO seems to be likely. In this sense, our data indicated that being born in a rural area and having exposure to biomass during early childhood in a patient with COPD were significant risk factors for the development of ACO in later life. However, our results also showed that exposure to environmental tobacco smoke (ETS) was as high as 60% in all groups regardless of underlying respiratory diseases. History of childhood asthma and allergic rhinitis have also been shown to be risk factors for the development of ACO in adulthood. Moreover, patients with ACO were reported to have allergies mostly, like patients with asthma (22,27). Suggesting this, our study showed that most patients with ACO had allergies, as demonstrated by positive

skin prick tests. Therefore, atopy should be evaluated in at-risk patients with COPD, particularly those with childhood asthma or allergic rhinitis. Morgan et al. have investigated the prevalence and risk factors of ACO in a total population of 11.900 patients in low- middle-income countries (28). The prevalence of ACO has been reported as 3.8%. Similar to our data, the authors have found that biomass exposure, smoking history, and low education levels are risk factors for having ACO. The rate of ACO among patients with COPD was high (43.8%), which was also comparable with our data. Sex features, spirometric measures, and BMI features were also similar to our data.

Concerning comorbidities, patients with ACO have been reported to be more likely to have multiple comorbidities, including obesity, hypertension, and gastroesophageal reflux disease in previous studies (5,29). According to primary care research involving 2165 patients, patients with ACO exhibit common comorbidities such as diabetes (53%), cardiovascular disease (36%), hypertension (30%), eczema (23%), and rhinitis (21%) (5). When compared with the COPD-only sample, people with ACO are less likely to have rhinitis and more likely to have chronic renal disease than those with asthma alone. In our study, the patients with ACO had a similar rate of chronic rhinosinusitis, nasal polyps, and NSAID hypersensitivity, like in asthma, and a similar rate of ischemic heart disease, like in COPD. Therefore, patients with either asthma or COPD with such comorbidities should be carefully reviewed for ACO.

ACO has been defined as a more severe disease than asthma alone. Our results supported this in several ways. First, in our series, the patients with ACO had severe and progressive symptoms of dyspnea, phlegm, and dyspnea on exertion, which were similar to those with COPD and different from those with asthma. Secondly, pulmonary functions also showed that these patients had severe airway obstruction like patients with COPD, but also had highly variable airway obstruction like in asthma. Radiologic features mostly showed air trapping, which was suggestive of airway obstruction. Therefore, the presence of these clinical findings and suggestive laboratory results, especially in patients with asthma, should be regarded as a warning sign for the future development or the presence of ACO.

Laboratory techniques could provide more detailed data for the diagnosis of ACO. In previous studies, patients with ACO were reported to have more bronchial wall thickness and more air trapping or emphysema on computed tomography scans of the thorax (30,31). Our results also provided similar data.

ACO is associated with more progressive loss of airway function than asthma and COPD alone (32,33). Importantly, our data showed that the annual

FEV1 decline was higher in patients with ACO. Another study from the United States of America also

suggested that ACO was associated with more severe asthma and COPD, as well as decreased lung function compared with COPD or asthma alone (7). This severe airway obstruction has been associated with airway inflammation (34). This is possibly the most important reason why patients with COPD or asthma should be evaluated for the presence of ACO. These results, therefore, suggest that awareness about ACO, particularly in at-risk groups, may be associated with better management and prevention of the progression of the disease.

Our study has some strengths and limitations. Given the large population of the included regions and the size of the study population, the data obtained should be considered valid concerning the documentation of risk groups for the development of ACO in our country. However, for the evaluation of

FEV1 decline, most patients had two measurements a year apart. It would be preferable to follow the

patients for more than a year. This may be considered a limitation of our study.

In conclusion, this study showed that ACO was common among patients with asthma and especially among patients with COPD, and was associated with a more severe degree of airway obstruction and progressive loss of pulmonary function in comparison with asthma. Therefore, one should be aware of the existence of ACO in patients with asthma if they have advanced age, particularly male subjects, with fixed airway obstruction and a severe airway, with biomass exposure during childhood, and if they were raised in a rural area. Patients with COPD should also be reviewed for the possibility of ACO if the patient has atopy, eosinophilia, childhood allergic rhinitis/ asthma, and sinusitis or NSAID hypersensitivity. Strategies to improve the awareness of physicians about ACO and its diagnostic criteria should be regularly implemented and relevant treatment should be started because it appears to be associated with progressive loss of pulmonary function. Moreover, considering high exposure to ETS during childhood in all groups, strategies to reduce passive exposure, particularly during childhood, seems to be urgently necessary for national policies.

**Ethical Committee Approval:** This study was approved by the Ankara University Faculty of Medicine (Decision no: 12-396-12, Date: 23.07.2012).

## CONFLICT of INTEREST

The authors declare that they have no conflict of interest.

## AUTHORSHIP CONTRIBUTIONS

Concept/Design: GEÇ Analysis/Interpretation: AHE Data acqusition: All of authors Writing: GEÇ, ÖA, EŞ

Clinical Revision: All of authors Final Approval: All of authors

## REFERENCES

1. *
  Bleecker ER. Similarities and differences in asthma and COPD. The Dutch hypothesis. Chest 2004; 126(2 Suppl): 93S-5S; discussion 159S-61S. https://doi.org/10.1378/ chest.126.2_suppl_1.93S*
2. *
  Global Initiative for Asthma. Global strategy for asthma prevention and treatment. Available from: https://ginasth- ma.org/wp-content/uploads/2019/11/GINA-GOLD- ACOS_2015.pdf.
  *
3. *
  Wurst KE, Kelly-Reif K, Bushnell GA, Pascoe S, Barnes N. Understanding asthma-chronic obstructive pulmonary disease overlap syndrome. Respir Med 2016; 110: 1-11. https://doi.org/10.1016/j.rmed.2015.10.004*
4. *
  Guerriero M, Caminati M, Viegi G, Senna G, Pomari C. Prevalence and features of asthma-chronic obstructive pulmonary disease overlap in Northern Italy general pop- ulation. J Asthma 2019; 56(1): 27-33 https://doi.org/10.1 080/02770903.2018.1424190*
5. *
  Krishnan JA, Nibber A, Chisholm A, Price D, Bateman ED, Bjermer L, et al. Prevalence and characteristics of asth- ma-chronic obstructive pulmonary disease overlap in routine primary care practices. Ann Am Thorac Soc 2019; 16(9): 1143-50. https://doi.org/10.1513/ AnnalsATS.201809-607OC*
6. *
  Kumbhare S, Pleasants R, Ohar JA, Strange C. Characteristics and prevalence of asthma/chronic obstruc- tive pulmonary disease overlap in the united states. Ann Am Thorac Soc 2016; 13: 803-10. https://doi. org/10.1513/AnnalsATS.201508-554OC*
7. *
  Mendy A, Forno E, Niyonsenga T, Carnahan R, Gasana J. Prevalence and features of asthma-COPD overlap in the United States 2007-2012. Clin Respir J 2018; 12(8): 2369- 77. https://doi.org/10.1111/crj.12917*
8. *
  Jo YS, Hwang YI, Yoo KH, Kim TH, Lee MG, Lee SH, et al.; Korean Asthma Research Group & KOCOSS cohort. Comparing the different diagnostic criteria of Asthma- COPD overlap. Allergy 2019; 74(1): 186-9. https://doi. org/10.1111/all.13577*
9. *
  Sin DD, Miravitlles M, Mannino DM, Soriano JB, Price D, Celli BR, et al. What is asthma-COPD overlap syndrome? Towards a consensus definition from a roundtable discus- sion. Eur Respir J 2016; 48(3): 664-73. https://doi. org/10.1183/13993003.00436-2016*
10. *
  Cosio BG, Soriano JB, López-Campos JL, Calle-Rubio M, Soler-Cataluna JJ, de-Torres JP, et al.; CHAIN Study. Defining the Asthma-COPD overlap syndrome in a COPD cohort. Chest 2016; 149(1): 45-52. https://doi. org/10.1378/chest.15-1055*
11. *
  Plaza V, Álvarez F, Calle M, Casanova C, Cosío BG, López- Viña A, et al. Consenso sobre el solapamiento de asma y EPOC (ACO) entre la Guía española de la EPOC (GesEPOC) y la Guía Española para el Manejo del Asma (GEMA). Arch Bronconeumol 2017; 53: 443-9. https:// doi.org/10.1016/j.arbres.2017.04.002*
12. *
  Alshabanat A, Zafari Z, Albanyan O, Dairi M, Fitz Gerald JM. Asthma and COPD overlap syndrome (ACOS): A systematic review and meta analysis. PLoS ONE 2015; 10(9): e0136065. https://doi.org/10.1371/journal. pone.0136065*
13. *
  Ding B, Enstone A. Asthma and chronic obstructive pul- monary disease overlap syndrome (ACOS): Structured literature review and physician insights. Expert Rev Respir Med 2016; 10(3): 363-71. https://doi.org/10.1586/1747 6348.2016.1144476*
14. *
  Nielsen M, Barnes CB, Ulrik CS. Clinical characteristics of the asthma-COPD overlap syndrome - a systematic review. Int J COPD 2015; 10: 1443-54. https://doi. org/10.2147/COPD.S85363*
15. *
  Miravitlles M. Diagnosis of asthma-COPD overlap: Is it possible a global definition. Pulmonology 2018; 24(3): 143-5. https://doi.org/10.1016/j.pulmoe.2018.02.002*
16. *
  Chambliss JM, Sur S, Tripple JW. Asthma versus chronic obstructive pulmonary disease, the Dutch versus British hypothesis, and role of interleukin-5. Curr Opin Allergy Clin Immunol 2018; 18: 26-31. https://doi.org/10.1097/ ACI.0000000000000409*
17. *
  Yorgancıoğlu A, Türktaş H, Kalaycı O, Yardım N, Buzgan T, Kocabaş A et al. The WHO global alliance against chronic respiratory diseases in Turkey (GARD Turkey). Tuberculosis Thorax 2009; 57(4): 439-52.
  *
18. *
  Global Initiative for Asthma. Global strategy for asthma prevention and treatment. Available from: https://ginasth- ma.org/wp-content/uploads/2019/01/2014-GINA.pdf.
  *
19. *
  Global Strategy for the Diagnosis. Management and pre- vention of COPD, Global Initiative for Chronic Obstructive Lung Disease (GOLD) 2014. Available from: http://www. goldcopd.org (Accessed date: 08.04.2022).
  *
20. *
  de Marco R, Accordini S, Antonicelli L, Bellia V, Bettin MD, Bombieri C, et al. The gene-environment ınteractions in respiratory diseases (GEIRD) project. Int Arch Allergy Immunol 2010; 152(3): 255-63. https://doi. org/10.1159/000283034*
21. *
  Wheaton AG, Pleasants RA, Croft JB, Ohar JA, Heidari K, Mannino D, et al. Gender and asthma-chronic obstructive pulmonary disease overlap syndrome. J Asthma 2016; 53(7): 720-31. https://doi.org/10.3109/02770903.2016. 1154072*
22. *
  van Boven JFM, Roman-Rodriguez M, Palmer JF, Toledo- Pons N, Cosio BG, Soriano JB. Comorbidome, pattern abd impact of Asthma-COPD overlap syndrome in real life. Chest 2016; 149(4): 1011-20. https://doi.org/10.1016/j. chest.2015.12.002*
23. *
  Hardin M, Silverman EK, Barr RG, Hansel NN, Schroeder JD, Make BJ, et al.; COPD Gene Investigators. The clinical features of the overlap between COPD and asthma. Respir Res 2011; 12: 127. https://doi.org/10.1186/1465-9921- 12-127*
24. *
  Zeki AA, Schivo M, Chan A, Albertson TE, Louie S. The asthma-COPD overlap syndrome: A common clinical problem in the elderly. J Allergy (Cairo) 2011; 2011: 861926. https://doi.org/10.1155/2011/861926*
25. *
  Sevimli N, Yapar D, Türktaş H. The prevalence of asth- ma-COPD overlap (ACO) among patients with asthma. Turk Thorac J 2019; 20(2): 97-102. https://doi. org/10.5152/TurkThoracJ.2018.18055*
26. *
  de Marco R, Pesce G, Marcon A, Accordini S, Antonicelli L, Bugiani M, et al. The Coexistence of asthma and chron- ic obstructive pulmonary disease (COPD): Prevalence and risk factors in young, middle-aged and elderly people from the general population. PLoS ONE 2013; 8(5): e62985. https://doi.org/10.1371/journal.pone.0062985*
27. *
  Barrecheguren M, Román-rodríguez M, Miravitlles M. Is a previous diagnosis of asthma a reliable criterion for asth- ma-COPD overlap syndrome in a patient with COPD? Int J COPD 2015; 10: 1745-52. https://doi.org/10.2147/ COPD.S87025*
28. *
  Morgan BW, Grigsby MR, Siddharthan T, Chowdhury M, Rubinstein A, Gutierrez L, et al. Epidemiology and risk factors of asthma-chronic obstructive pulmonary disease overlap in low- and middle-income countries. J Allergy Clin Immunol 2019; 143(4): 1598-606. https://doi. org/10.1016/j.jaci.2018.06.052*
29. *
  Mart MF, Peebles RS Jr. Asthma-chronic obstructive pul- monary disease overlap syndrome. Curr Opin Immunol 2020; 66: 161-6. https://doi.org/10.1016/j. coi.2020.10.006*
30. *
  Suzuki T, Tada Y, Kawata N, Matsuura Y, Ikari J, Kasahara Y, et al. Clinical, physiological, and radiological features of asthma-chronic obstructive pulmonary disease overlap syndrome. Int J COPD 2015; 10: 947-54. https://doi. org/10.2147/COPD.S80022*
31. *
  Gao Y, Zhai X, Li K, Zhang H, Wang Y, Lu Y, et al. Asthma COPD overlap syndrome on CT densitometry: A distinct phenotype from COPD. COPD 2016; 13(4): 471-6. https://doi.org/10.3109/15412555.2015.1102874*
32. *
  Sin DD. Asthma-COPD overlap syndrome: What we know and what we don’t. Tuberc Respir Dis 2017; 80: 11-20. https://doi.org/10.4046/trd.2017.80.1.11*
33. *
  Lange P, Colak Y, Ingebrigtsen TS, Vestbo J, Marott JL. Long term prognosis of asthma, chronic obstructive pulmonary disease, and asthma-chronic obstructive pulmonary dis- easeoverlap in the Copenhagen City Heart study: A pro- spective population- based analysis. Lancet Respir Med 2016; 4: 454-62. https://doi.org/10.1016/S2213- 2600(16)00098-9*
34. *
  Uzan GC, Borekci S, Doventas YE, Koldas M, Gemicioglu
  *

*
B. The relationship between inflammatory markers and spirometric parameters in ACOS, Asthma, and COPD. J Asthma 2020; 57(12): 1273-9. https://doi.org/10.1080/0 2770903.2019.1652644*

